# Editorial: Languages as Adaptive Systems

**DOI:** 10.3389/fpsyg.2019.01555

**Published:** 2019-07-17

**Authors:** Umberto Ansaldo, Enoch Oladé Aboh

**Affiliations:** ^1^Department of Linguistics, The University of Hong Kong, Hong Kong, Hong Kong; ^2^University of Sydney, Sydney, NSW, Australia; ^3^Department of Linguistics, Universiteit van Amsterdam, Amsterdam, Netherlands

**Keywords:** language, linguistics, complexity, adaptive, ecology, evolution, diversity, variation

One of the most puzzling features of human language and its use lies in its diversity of forms. From one (or a few) alleged human language(s) spoken some 100,000 years ago, we have today at least 7,000 different languages spoken or signed.

As we know well from the study of classical languages (e.g., Greek, Latin, Sanskrit), every language changes over time. Accordingly, a fundamental question in the language sciences is: how and why does this change happen? Are there any significant constraints? When we think of the growth of the human population, the waves of colonial conquests throughout history, and the language contact situations they bring, the common estimation of 7,000 languages suggests a paradox: linguistic systems change continuously under the agency of their speakers/signers, yet the number of language types described by typological books appears relatively small. The paradox dissolves, however, if we assume that learners develop adaptive linguistic systems based on their specific ecologies, and that these systems follow patterns determined by human cognition. Understanding the flexibility as well as the range of constraints in our linguistic systems—and how they vary—is part of understanding our human nature.

The papers in this volume approach these issues from a multidisciplinary perspective and through a variety of empirical and experimental sources of evidence.

Progovac et al. approach the question of the origin of diversity based on a functional MRI experiment investigating differential patterns of brain activation during processing of sentences with “minimal” vs. “fuller” syntactic structures. Their findings support previous proposals that early stages in language evolution involved basic syntactic computations emerging from a diffused cerebral network, which provided the foundation for further elaborate and complex syntactic computation.

The question of order within diversity is addressed in Ghyselen and De Vogelaer from a sociolinguistic perspective, aiming to understand the extent of systematicity within what appears as complex variation. Their corpus-based study of the uses of spoken Dutch varieties in West Flanders (Belgium) proposes that the heterogeneity observed in dialectal variation can be reconciled with a structured gradient of dialect continua.

Another perspective on unity within diversity is presented in Couvee and Pfau, based on the study of Sign Language of the Netherlands. The authors show that the distribution of certain verbs—namely GO, GIVE, TAKE, and CALL—in combination with other lexical verbs follows parallel patterns to Serial Verb Constructions (SVC) in spoken languages. As has been reported for SVC in spoken languages, they show that the verbs from this closed class can also develop into various grammatical markers such as (i) GO functioning as a future tense marker, and (ii) GIVE functioning as a light verb. Such broad cross-modality characterizations of change indicate possible evolutionary preferences that provide “order” within variation.

The search for constraints—or “inhibitors”—is brought into the auditory domain by Havenhill and Do. This study is based on production and perception experiments of specific vowels in the Northern Cities Vowel Shift of the Great Lakes region of the United States. The results reveal an interaction of articulatory variation and audiovisual speech perception. The authors therefore not only confirm that inhibitors are found in sound change, but also that visual cues can shape phonological systems through misperception-based sound change.

How linguistic complexity may have evolved is the topic of the opinion piece by Benítez-Burraco and Kempe. Based on the assumption that the human brain has not changed substantially since our origins, they argue that neuro-anatomical and concomitant behavioral changes observed in modern humans are largely manifestations of human self-domestication. The authors propose that social behaviors such as parenting and teaching are at the core of cultural and language transmission processes that favor the emergence of complex cognitive capacities.

Plat et al. investigate the question of how first language acquisition affects subsequent language learning experimentally in Dutch speakers of English as L2. A word naming task conducted on these speakers suggests that first language word processing might be more automated than subsequent language processing. The authors remark, however, that generalizing from monolingual studies to bilinguals requires caution, as the multilingual “individual's adaptability to task, circumstance and environment is underestimated in such direct comparisons.”

Language mixing is a well-known yet not entirely understood aspect of languages as adaptive systems. Alexiadou and Lohndal investigate speakers' adaptability to various linguistic ecologies and how this may affect their speech. Drawing on Distributed Morphology, they show that, while a range of structural constraints to word-internal mixing in various typologically different languages (e.g., Cypriot Greek-English, English-Norwegian, Greek-English, Greek-German, Spanish-German) can be identified, these vary dramatically depending on the syntactic domain in which they occur, as well as the structural relationships between the languages in contact.

De Clerq and Haegeman discuss original data from speakers of the Ghent dialect who use a pleonastic particle *die* in constructions in which the first constituent of a main clause is an adverbial adjunct, followed by the particle *die*, followed by the finite verb. Such superficial V3 patterns seem very atypical of a West Germanic language, given the rigid verb second (V2) rule commonly observed in these languages. The authors, however, demonstrate that superficial V3 patterns in the Ghent dialect represent a structural subtype of classical V2, in which the particle *die* occupies a slot that would otherwise host a verbal element or a complementizer.

The papers in this volume aim to present a view of language as a complex system that adapts to its specific ecology. Human languages are used in a diverse range of ecologies determined by speakers/signers' interactions within and across communities. These ecologies are crucial for language acquisition because they provide the language learner with the input on which she will base her learning hypotheses. Since most learners in a community adapt to their particular ecology, partially determined by their social network, not all learners converge to the same adaptive systems. Hence the origin of language change.

## Author Contributions

All authors listed have made a substantial, direct and intellectual contribution to the work, and approved it for publication.

### Conflict of Interest Statement

The authors declare that the research was conducted in the absence of any commercial or financial relationships that could be construed as a potential conflict of interest.

